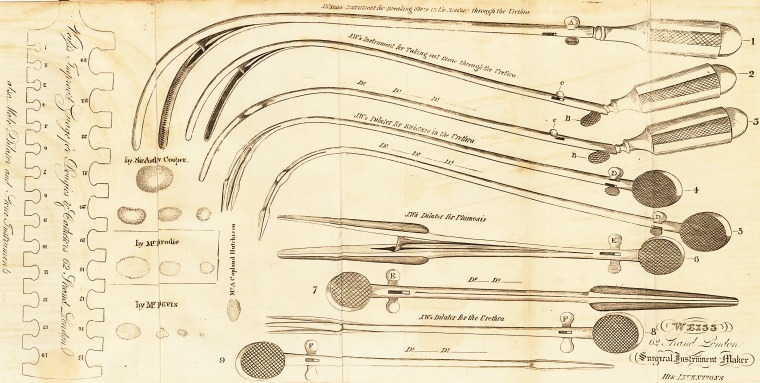# Extra Limites

**Published:** 1824-06-01

**Authors:** 


### XVI.
### EXTRA-LIMITES.

### I.
### MR. BATTLEY ON THE COMPONENTS OF OPIUM.

*To the Editors of the Medico-Chirurgical Review.*

Gentlemen,

In pursuing the object, agreeably to my promise, of showing the constituents of opium, I shall, in the first place, speak of that part or portion of this drug, which has been introduced into medical practice by the French, under the name of *Morphium*.

Twenty-six pounds (avoirdupois) of dry Opium imparted to distilled water twenty-three pounds, leaving a residuum weighing three pounds, when dried: this residuum or *refuse*, I apprehend to contain the *morphium*, and to the exposition of this fact, my present and immediate purpose is confined.

This residuum of three pounds, was macerated in a mixture consisting of fourteen pints of distilled water and two pints of strong acetic acid, for twelve hours, three times, and to the liquor when drawn off, Ammonia was added in excess, when a change to a creamy state ensued. The creamy substance was shortly precipitated, and being separated from the fluid, was washed repeatedly in distilled water, and when dried weighed 38 drachms 20 grains. This substance I apprehend to be *morphium, so called*, (impure) and when divided by means of boiling sulphuric æther and alcohol, was found to consist of:—

| | | Drachms. | Grains. |
|---|---|---|---|
| Resinous matter | | 15 | 7 |
| At the rate of 29 grains per drachm of the 38 drachms 20 grains. | Crystals or *morphium, so called* (pure) | 19 | 4 |
| | Ditto, less pure | 1 | 48 |
| Matter resembling earth, afterwards dissolved in a diluted solution of potassa (less 13 grains) | | 2 | 21 |
| | | 38 | 20 |

or 5 oz. avoirdupois and 100 grains.

Of the residuum of three pounds, 5 ounces remained suspended in the liquor, and 38 oz. in a fibrous greasy state, smell and taste unpleasant, peculiar to opium; this latter was boiled in alcohol twice, and being pressed, left in the cloth about 29 oz. having the appearance of calamita styrax, *free from the greasy appearance,* and nearly so *from the peculiar opium smell and taste.*

The alcohol thus boiled became of a deep brown colour, and on cooling, a tenacious waxy matter adhered to the sides, and bottom of the vessel;—in this waxy matter the peculiar smell and taste before noticed, prevailed to an intense degree. The waxy matter weighed about $7\frac{1}{2}$ oz., was highly combustible, forming compounds with oil and turpentine, and of a specific gravity somewhat exceeding water. By means of Papin's still, the alcohol was then brought over, and left about $2\frac{1}{4}$ oz. of resinous matter, partaking strongly of the taste and smell of Opium:—thus,

| | Oz. | Qrs. |
|---|---|---|
| Waxy substance . . . . . . . . . . . . . . | 7 | 2 |
| Resinous matter . . . . . . . . . . . . . . | 2 | 1 |
| Appearing like calamita styrax . . . . . . . | 29 | 0 |
| | 38 | 3 |

being an increase of $\frac{1}{4}$ oz. which I apprehend to arise from the retention of moisture by the resinous and waxy matter.

The 29 oz. appearing like styrax, by the addition of diluted solution of potassa became gelatinous and greatly increased in bulk, and being dried at a temperature of 150°, formed a substance which, when broken, exhibited a shining fracture.

Recapitulated, the residuum of three pounds is accounted for as follows:—

| | Oz. | Qrs. | Grains. |
|---|---|---|---|
| *Morphium, so called,* (impure) 38 drs. 20 grs. or | 5 | 0 | 100 |
| Remained suspended in the first process . . | 5 | 0 | 0 |
| Last above mentioned . . . . . . . . . | 38 | 3 | 0 |
| | 48 | 3 | 100 |

I now proceed to show a similar result from the residual matter of Tincture of Opium, Tincture-bottoms. Of this matter, when perfectly dried, one pound was macerared in a mixture of strong acetic acid and distilled water for 12 hours. The maceration was repeated twice, and again twice in a similar mixture, at a temperature of about 150°, and to the liquor when drawn off, ammonia was added in excess;—a change to a creamy state ensued, as in the first mentioned experiment and the creamy substance was in like manner washed and dried, and weighed

10 drachms 28 grains ;—of this quantity, 8 drachms, 45 grains, were divided by means of boiling æther and alcohol, and consisted of :—

| | Drachms. | Grains. |
|---|---|---|
| Resinous matter . . . . . . . . . . . . . . | 4 | 6 |
| At the rate of 28 grains per drachm of the 8 dra. 45 gns. } Crystals, or *morphium, so called,* (pure) . . . . | 4 | 4 |
| Matter resembling earth . . . . . . . . . | 0 | 56 |
| | 9 | 6 |

being an increase of 21 grains, which I apprehend to arise from the spirit detained in the extract.

Results nearly similar to those already mentioned, were obtained by boiling alcohol, from the remaining portion of the residual matter; that is to say, the substance having the appearance of calamita styrax, the waxy substance, and resinous matter.

The results were also similar, so far as the experiment was carried, from 10 lbs. of the residuum or *refuse* of Opium, subjected three times to boiling alcohol, viz :—

| | Oz. | Qrs. | Gr. |
|---|---|---|---|
| Resinous matter . . . . . . . . . . . . | 15 | 0 | 0 |
| A Crystalline mass . . . . . . . . . . . | 19 | 3 | 20 |

This mass, when reduced by solution, and by the separation of the waxy and other matter, by means of boiling æther and alcohol, re-formed in crystals perfectly similar to the morphium, 19 drachms, 4 grains, and 4 drachms, 4 grains, resulting from the two several processes first mentioned, and weighed 62 drachms, 52 grains.

I shall in your next number, with your permission, advert again to Morphium, and to a fluid intimately combined with the waxy and resinous matter, and closely connected with the peculiar smell and taste of Opium; and then proceed to show the constituents of the twenty-three pounds imparted to distilled water (part of twenty-six pounds) as first above mentioned, but I must not now conclude without stating that laudanum, Tincture of Opium, does not contain any, or if any, only a very small portion of *Morphium,* (so called) and recent observation tends to confirm an opinion which I have long entertained, namely, that *Morphium* does not partake of the sedative properties of Opium, in more than a very limited degree, if at all.

I am, Gentlemen,

Your obedient Servant,

RICHARD BATTLEY.

Fore Street, May 15, 1824.

### II.

### DR. SANDERS ON THE TREATMENT OF PHTHISIS.

*To the Editor of the Medico-Chirurgical Review.*

Suum cuique.

SIR,—The 13th Number of your excellent Journal, dated June 1823, contains two articles, for my taking notice of which, when you see my annotations, the reason will be obvious, as well as the apology.

The one which claims priority is, *The Tonic Treatment of Phthisis, p.* 189. It is stated that, "in a paper by Dr. John Hume, you have seen many interesting remarks on that scourge of our country, Phthisis Pulmonalis, and also an exposé of Dr. Stewart's plan, which is not generally known to the profession;" and (p. 190) Dr. Hume says, "that the greater part of his plan is borrowed from Dr. Stewart, who was led to the *peculiar* practice, which he has adopted in consumption, from *reflecting attentively* on some of Dr. Gregory's statements, when lecturing on that disease."

Dr. Hume has made two important omissions, the one is, that he has not favored us with those statements, and those critical points in them, which exclusively drew the attention of Dr. Stewart; our dull comprehension needs such assistance, since we never could have expected the tonic plan to receive its first impulse from the quarter assigned. In the understanding of Dr. Gregory, during the 40 years, or more, that he was professor; in mine, while I attended either his class or the sick along with him; and in that of his other pupils successively; his statements led to opinions and prescriptions the very antipodes of the Tonic Method. We cannot, therefore, readily consent, that our illustrious preceptor, in sound mind, gave out *oracular* sentences, unintelligible to himself, and to all human beings besides, till Dr. Stewart began to exert *his* faculties of reflection. In ancient days, the Delphic Pythoness, in frantic raving, emitted responses fraught with fate, and none but those of the priesthood could unveil the secrets of the mystic diction; perhaps Dr. Stewart, having two attributes of Apollo, divinity and physic, is in our days, the only one possessed of the sacred gift, with this superiority over his prototypes, that he discovers mystery and the rudiments of momentous change, where, to every one else, there appears only plain and undisguised narration!

To be serious, I heard years ago, that something stated by Dr. Gregory suggested the tonic plan of treatment, and I suspect, that Dr. Hume has merely repeated this report without any direct authority. It may be asked, why did not Dr. Stewart disclose, whence he had the method ascribed to him, and contradict the account published by Dr. Hume? I answer, that a man may have motives for concealing what is true; though he would not incur the guilt and reproach of wilful and deliberate falsehood.

Dr. Hume has not supplied *data* by which we could solve any question concerning the origin of the plan which he professes to have borrowed. I have remarked, that he had made two important omissions, the one relative to the alleged statements I have considered; the other is this, he has not told us at what time the paroxysm of inventive cogitation attacked Dr. Stewart. Was it before, or was it after, the Tonic Method was brought under the review of the Medical Society of Edinburgh, in which the reverend physician was one of the presidents? If before, why did he conceal, or rather why did he not seize an occasion so suitable for declaring his sentiments? If after, of what avail was his attentive reflection? I will endeavour to supply the deficiencies of Dr. Hume, by giving both statements and dates. In fine, I doubt not I shall make it evident, *when, where, and from whom Dr. Stewart borrowed the plan entire in principle and in application.*

For several years previous to 1802, when I was admitted a member of the Medical Society, I had been occupied with the investigation of the Nature and Treatment of Phthisis Pulmonalis; being satisfied myself, and believing that I could satisfy others, that many would have been saved, and the sufferings of many more alleviated, or their lives prolonged, by a kind of management opposite to that then prevalent, I introduced *The Tonic Treatment of Phthisis* to the consideration of that Society *where Dr. Stewart had long signalized himself as an orator, and, be it remembered, long after he had attended the Lectures of Dr. Gregory;* yet, even at that time, so little impression had any former statements made on his retrospective intellect, that he added his voice to theirs, who denounced the Tonic Treatment as a daring innovation. It was not a topic of private conversation amongst a few, it roused the minds of all who attended the meetings, or, at least, who took any concern in the business for which they were held; contention had not been so hot from the time of the Cullenians and Brunonians; some said, that my notions were fanciful; others, that my conclusions were premature; some maintained, that the system was neither new nor original; and, not a few, pronounced it presumption in so young a man, to impugn doctrines and proceedings so long approved and taught by eminent and learned men, whose reputation had increased with their years. I replied, that, if my notions were fanciful, I appealed to facts; if my conclusions were premature, I submitted to their deliberate examination, both premises and conclusions; with regard to novelty or originality, I aimed at neither; utility was the end of my exertions; with regard to derogating from lessons and conduct of high sanction, this was an objection of equal force against every species of improvement; that while I had the utmost respect for our predecessors and seniors, I bowed to no authority in science, but that of truth. Is not, said I, our knowledge of Phthisis confessedly very defective? and does the almost uniform failure of the means commonly employed, not demand serious and patient enquiry? Science is not to be advanced by plausible arguments, nor errors to be corrected by inveterate prejudices, yet by no other weapons, am I here assailed. You admit, that the plan of research which I have chosen, is unexceptionable, and that the results, if they were to be relied on, are of great value; ought you not then, to dispassionately ascertain whether there is any inaccuracy in the observations, or imperfection in the statements, rather than hastily reject those facts and views, which, even in your own judgment, if verified, would prove of such importance?

My opponents found it easy to reiterate their assertions, but not so, to refute my reasoning; in consequence, a small party spontaneously arose in favour of what were in ridicule called "*The New Doctrines;*" disputation became more animated and better sustained, a certain degree of interest was excited, and the controversy was no longer confined within the circumference of our Hall; the students dreamed, and it was only a dream, that the professors were offended; the sycophantish showed themselves now more noisily hostile, and the timid gave up or shunned my acquaintance, lest it should affect their graduation; some, professing friendship, admonished me no longer to agitate such subjects, as they raised against me a host of enemies, and truly, in these discussions, commenced professional antipathies which continue to this day. All this while Dr. Stewart afforded me no assistance; he gave no utterance to *his attentive reflections.*

But I am not one of those that can soon be deterred from vindicating what, I am convinced, is right. I induced several of my fellow members to visit the patients along with me; melioration or recovery imparted confidence in the means advised; and, when any case terminated fatally, the causes of failure were anatomically explored. The events fully confirmed my statements. In this manner, I procured a mass of evidence not to be resisted. At first, there was not one to second; now, scarcely one had the hardihood to oppose.

By this time, from 20 to 30 gentlemen had united their observations with mine, and the Tonic Treatment began to acquire something like attention. I do not believe, that one listened to these discussions whose practice is not more or less regulated by what he then heard; nor do I know above one or two in this city, who persevere in the old method of emaciation.

After my inquiries had undergone this severe scrutiny for two or three years, I presented a compendium of them in a paper, which stands on the records of the Society for 1805-6. This essay was the outline of a work which I had ready for the press in 1806, and which was published in January 1808, intitled "*Treatise on Pulmonary Consumption, in which a New View of the Principles of its Treatment is supported by Original Observations on every Period of the Disease, &c.*"

Within this period it was, that Dr. Stewart began to try the Tonic Method. Though he had been a pupil of Dr. Gregory, and a speaking member of the Society long before 1802, he never imparted the slightest idea of the Tonic Treatment; and, when brought forward by me, it was so foreign to his manner of thinking, that he was one of my most formidable antagonists; nor of a different spirit were his essays, as the records of the institution testify; he there evinces no deviation from the common routine. He neither spoke, wrote, nor acted according to the Tonic Plan, as far as we have been able to learn, till two years after he had been made acquainted with it, in all the amplitude of detail. How did the impressions made on him by Dr. Gregory lie so many years dormant? What, I ask again, was the necessity of his *attentively reflecting*, to devise a system that was formed for him, and explained to him, accompanied by all the facts by which it was supported; on whom could he flatter himself that such a fable would impose; or how could he fancy that such an imposture would escape detection?

Truth gradually undermines the prepossessions of her adversaries, so it was, I imagine, with Dr. Stewart, in defiance of himself, the Tonic Method had gained upon his thoughts; till an occurrence which I shall relate, gave it the ascendancy. Within the period above specified, he visited where a lady was under the old regime for Phthisis Pulmonalis; the disease proceeded uncontrolled, and her state was pronounced hopeless. Now he determined that nothing possible should be left unattempted. What was esteemed *the best treatment* having failed, he thought she could *but die*, under that which he had considered *the worst*. "A doubtful remedy is better than none." Instead of every privation, he ordered her nutritious diet, air, and exercise, sponging with cooling liquids to allay morbid heat; tonic medicines to strengthen the stomach and bowels; every thing calculated to invigorate the body and exhilarate the mind; in short, all the parts of the new mode, which was still sounding in his ears. His wishes were realized; the lady recovered, and her relations extolled him to the skies; other patients of rank applied, fortune did not desert the practitioner. Why, when elated with success, he did not tell to whom he was indebted, it is not for me to conjecture. I recollect well, that it was a favorite theme with Dr. Stewart, "that mind is independent of matter," but he was not so conscious then, perhaps, of the power of the precious metals!

It may be very true, as you say, that "Dr. Stewart's plan is not generally known to the profession," or, in other words, it is not generally known, that the Method of Treating Phthisis adopted by Dr. Stewart, is precisely that which I published about 16 years ago, and that the same Method has been practised by myself, and known to many of the profession nearly 20 years. The preface, the narrative, and the cases, with their dates, will establish incontrovertibly all that I have here written. You have only to open the small volume at the title, treatment, you will see the system fully developed; the very principles and illustrations of the plan; and those passages which you have quoted, you will there recognize nearly *verbatim et literatim*. Dr. Stewart, I say again, would hardly ascribe to Dr. Gregory, or claim for himself, the plan for which Dr. Hume unknowingly gives him credit; were, which I trust not, his disposition so despicable, yet prudence would prevent him, since he is aware, that there are many practitioners and teachers of medicine in this city, and physicians and surgeons in every quarter of the globe, equally acquainted with the facts, though they have not used them so advantageously.

Let Dr. Stewart and Dr. Hume settle this affair between themselves, at their leisure; and if this communication excite uneasy emotions any-where, let blame devolve on whom it ought; every man who assumes what is not his own, or who prefers emolument to integrity, must bear the consequences. This it is the duty of a reverend gentleman to inculcate, but really the misfortune of any one to experience.

I assure Dr. Stewart, however, that in preferring my claim, I do it without animosity or resentment; he has been of service, and so far I thank him; the first Phthisical Patients whom he had under his care, were persons of distinction; his success confirmed the practice, and their influence gave it celebrity; he, therefore, afforded me no inconsiderable assistance to substitute a rational and salutary, instead of an empyrical and dangerous, manner of thinking and acting in that horrid disease, which you have well named "the scourge of our country."

I dare say, Mr. Editor, you are tired of this exposé, and so am I;—just a word or two more before we part. "Suum cuique," the motto prefixed to this letter, implies a moral precept, which I am sure you wish to obey.

This historical sketch of the origin and progress of the *Tonic Plan*, will enable you to decide whose it is; and whether it deserves commendation, or the contrary, you will attribute to Dr. Stewart no other merit or demerit, than *that of borrowing, without acknowledgement, the system to which he adheres, in the treatment of Pulmonary Consumption.*

Yours truly,

JAMES SANDERS.

*Edinburgh, March* 1824.

---

### III.

### MR. WEISS'S SURGICAL INSTRUMENTS.

In publishing the Plates, with the descriptions and observations accompanying them, sent us by Mr. Weiss; we deem it a justice due to him, to take the opportunity of briefly enumerating all the various other instruments invented by him for surgical purposes, which are not already noticed, and which are taken from his Catalogue.

1. The Aneurism Needle for passing ligatures under deep-seated arteries, which enables surgeons to perform the tying of those arteries with facility, which was previously done with great difficulty.—Messrs. Kirby, Travers, and Brodie, bear testimony to the utility of this invention.

2. The Dilator of the Female Urethra, which dilates the urethra, with great ease, to an extent, that admits of the admission of a fore-finger to feel the stone, and of a pair of forceps to extract it. By these means, very large stones have been extracted from the female bladder,

Surgical Instrument Maker

His Inventions by Sir A. Cooper, Mr. J. H. Green, Mr. Phillips, Mr. Brodie, and Dr. A. Ramsay.

3. A Hernia Knife, so contrived, that its cutting edge may be covered at pleasure.

4. A new Speculum Ani aut Vaginæ, which possesses many advantages over the old ones. There is a plate of another Speculum, which Mr. Weiss considers an improvement on the above.

5. A newly invented Enema Syringe, which prevents the injection of atmospheric air with the lavement, and may be used in five different positions by the patient, without assistance.

6. An improved Cupping Apparatus, in which a globe vacuum is attached to the cupping glass, so that when the glass is applied to the part to be cupped, a cock is turned, and the air from the glass rushes into the vacuum; after which, the glass is removed from the globe, which is screwed on other glasses in succession, as often as the occasion requires.

7. The Female Urethra Dilator, further improved, so as to enable it to dilate in the direction of a straight line.

8. The Flexible Gum Catheters, constructed so as to retain the form of the urethra, originally given them in the manufacture. "They do not suffer (says Sir E. Home) from the urine in the bladder, or the mucus in the urethra," and no incrustations adhere to their highly polished surface.

There are some other inventions of Mr. Weiss, of minor importance, not noticed in the Catalogue.

It must strike every one, on the most superficial consideration, how much the reputation of English surgeons of the present era, has been upheld by Mr. Weiss's ingenuity, as his inventions have enabled them to perform operations and relieve the greatest human afflictions, in a manner, that will be the admiration of all posterity. He observes, that he has not a patent for any of his inventions, which, the profession must be aware, could only be accomplished and compleated at a great expence, and with much devotion of time. He, therefore, sanguinely anticipates (what he deserves) the patronage of the profession at large, and as "the first professional gentlemen go to his house, when any idea strikes them that their surgical instruments are defective and can be improved," in order that his ingenuity may be rendered available, it is only fair to suggest, that they should "go to his house" and patronize him, when they want instruments not of his invention, or drawn from his ingenious mental store.

"*Explanation of the Instruments delineated in the Plate published in this Number of the Medico-Chirurgical Review.*

"No. 1. Take hold of the flat part, A. and, by turning the handle, you can open it to its full extent. As soon as you feel you have the stone between the blades, turn back the handle, and the strength of the springs will break the stone.

" No. 2. The thumb, when pressed upon the upright, B, will open the instrument; C, is a small screw to be taken out for the purpose of further extending the instrument in case it should grasp a stone too large to be extracted through the Urethra.
" No. 3. Shews the instrument closed.
" No. 4. An instrument which has given much satisfaction to Mr. Guthrie. To open it as described in the plate, hold it with the finger and thumb at D., and turn the handle to the right: turn it to the left, and it will shut as in fig. 5.
" No. 6 & 7. An instrument much approved of and recommended by Mr. Brodie for Phimosis, which opens and shuts like the last instrument.
" No. 8 & 9. A small instrument for dilating the Urethra.
" Mr. Weiss having made several useful inventions, thinks he is entitled to lay before the public a complete guage which may be used for his instruments as also for bougies and catheters. This guage possesses a great advantage over Mr. Smith's for metallic bougies, which only graduates from one to twelve, being better divided and having more sizes. This will be of great advantage to gentlemen in the country; he therefore intends to sell them at the very moderate price of 6s. 6*d*. by which means they will come into general use, and he flatters himself the profession will approve of his plan and give it a preference, as there are at present so many different guages."

### IV.
### SURGICAL ENGRAVINGS.

### PROSPECTUS OF A SERIES OF ENGRAVINGS,

DESIGNED AS

*Practical Illustrations of the Surgical Anatomy of the Blood-vessels, Nerves, and other important Parts divided in Amputation.*

By THOMAS ALCOCK, Surgeon.

THE difficulties occasionally experienced in finding and securing the arteries, and in avoiding the nerves, must have been observed by all who have witnessed many surgical operations. That these should occur is not surprising, when it is considered, that hitherto the Surgeon has possessed no aid in directing his attention to what he should readily find or certainly avoid, equal to that experienced by the traveller on referring to his map, or by the mariner in consulting his chart. It is the object of these Engravings to supply this deficiency, by exhibiting to the eye the actual and relative situation of all the principal arteries, veins, nerves, and other parts divided in amputation, at the various points usually selected for the performance of that operation; whilst the most essential circumstances are clearly explained by annexed notes of reference.

Each Plate comprises a finished coloured Engraving of the natural dimensions of the part of the body represented, to which are added an explanatory outline and copious notes of reference; so that each Plate forms a complete illustration (adapted to the advanced Student or to the practical Surgeon) of the amputation of that part of the limb represented, without depending upon any other of the series for explanation.

The first Plate represents a section of the leg, at the usual place of performing amputation below the knee, *i. e.* nearly one third the length of the tibia from its upper end; and may serve as a specimen of the work, and of the manner in which it is proposed to elucidate each subject.

From the care which has been bestowed to render these Engravings, with their explanatory references, accurate in all essential particulars, and subservient to actual practice, the Author trusts they may be found useful as guides, in directing the attention of the surgical Student, and as charts of reference to the experienced Surgeon.

Plate I. may be had of Messrs. Burgess and Hill, 55, Great Windmill Street, London, and of other medical booksellers, price 7*s.* 6*d.*; or mounted, 10*s.* 6*d.* A few proof Impressions, coloured under the Author's immediate direction, price 15*s.* each; or neatly mounted, fit for suspension in the surgery of the private practitioner, or in the operating rooms of hospitals, price 20*s.*

Plate II. illustrative of the amputation of the Leg by the flap-operation, is in forwardness, and will soon be published.

The operations on the Thigh, the Arm, the Fore-arm, as well as those at the Shoulder and Hip-joint, will be comprised in the remainder of the series.

Gentlemen who may be desirous to possess early impressions of the Plates, are requested to forward their names to the publishers, Messrs. Burgess and Hill, that the delivery of the copies may be in the order of subscription.

☞ *See outline of Plate No. 1, in this Review.*

---

Subscribers are informed that in consequence of the increased size of the Medico-Chirurgical Review, every two Numbers will hereafter form a Volume, of upwards of 500 pages, instead of putting four Numbers to the Volume.

---

N.B. Works have been received from Dr. Mills, Mr. Sandwith, and several others, too late for the Bibliographical Record of this quarter.

## Figures and Tables

**Figure f1:**